# Supplementary material for: Are carbon emissions trading and green financial instruments synergistic? -Comprehensive quantitative research based on content analysis
Source: PLoS One. 2024 Mar 7;19(3):e0298601. doi: 10.1371/journal.pone.0298601 (PMC10919601; doi:10.1371/journal.pone.0298601)
Supplement: S1 Data — (ZIP) [file pone.0298601.s001.zip › Data/地级市Z_F工作报告.zip!/╣π╢1⁄2/╘╞╕í╩╨/└┤╫╘╣1⁄2╓┌║┼í╛┬φ┐╦╩2╛▌═°í┐_2012.docx]

各位代表：

我代表市人民政府向大会作政府工作报告，请予审议，并请政协各位委员和其他列席人员提出意见。

本届政府任期工作回顾

本届政府任期的五年，是充满挑战和机遇的五年，也是我市全面实施“十一五”规划、启动“十二五”规划，推动经济社会实现加快发展的五年。市政府在省委、省政府和市委的正确领导下，在市人大及其常委会和市政协的监督支持下，带领全市人民，沉着应对国际金融危机等挑战，抢抓机遇、团结实干，圆满完成了市四届人大一次会议确定的各项目标任务。

（一）综合实力迈上新台阶。

预计2011年全市实现地区生产总值501.9亿元，比上年（下同）增长15%，比2006年增长1.2倍；人均生产总值2.1万元，增长13.9%，比2006年增长1.1倍；规模以上工业增加值183.2亿元，增长36.8%，比2006年增长2.2倍；地方财政一般预算收入29.8亿元，增长32.8%，比2006年增长1.6倍；全社会固定资产投资总额363.2亿元，增长30%，比2006年增长2.3倍；社会消费品零售总额168.6亿元，增长18.5%，比2006年增长1.3倍;外贸进出口总额13.5亿美元，增长0.7%，比2006年增长1.5倍；2011年11月末，金融机构本外币存款余额553亿元、贷款余额328亿元，分别增长13.6%和18%，分别比2006年增长1.2倍和2.1倍。主要经济指标增速在全省排位不断前移，2011年前三季度，规模以上工业增加值、地区生产总值、地方财政一般预算收入增速分别居全省第1位、第2位和第3位。

（二）转型升级实现新突破。

工业集聚发展态势基本形成。打造“五大”百亿特色产业集群取得突破性进展，各大产业均找到转型升级的有效载体。获批石材、不锈钢制品和电池等3个省级产业集群升级示范区。建成云浮国际石材博览中心，引进北方集团投资50亿元建设中国·云浮国际石材产业城。中材、华润、英坭等知名企业先后落户或增资扩产。新兴县不锈钢产业园、省市共建先进制造业（硫化工）产业基地建设加快推进。云浮发电厂C厂建成投产，云浮风电项目（罗定亚婆髻山和新兴天露山风电场）已动工建设，华润西江发电厂项目已上报国家能源局，云浮核电站（中广核、中电投）项目建议书已上报省发改委。

现代服务业快速发展。与四大商业银行和国开行签订授信总额556.8亿元融资协议，并已落实316亿元。大华农公司成功上市，温氏集团、新达公司成功发行企业债券。新培育限额以上商贸流通企业200家，建设一批“万村千乡”市场工程配送中心和农家店。2011年房地产开发投资21.5亿元，比2006年增长3.2倍。创建国家4A级旅游景区2个，举办3届禅宗六祖文化节和旅游文化美食节。2011年接待游客803万人次、旅游总收入87亿元，分别比2006年增长1.6倍和2.5倍。三网融合应用、物联网、文化创意、健康休闲等战略性新兴产业新业态初步形成。

农业农村经济较快发展。农业产业化组织发展到417个，其中省级农业龙头企业16家、市级以上农业龙头企业69家、农民专业合作社241个，带动27万农户增收致富。国家级农业龙头企业温氏集团三度入选广东百强民营企业前10位，并成为广东“千百亿”名牌培育工程企业，2011年销售收入突破300亿元。无公害农产品、绿色食品和有机食品生产基地发展到52个，认证产品分别发展到58个、23个和9个。郁南县10万亩沙糖桔生产基地、罗定市20万亩稻谷生产基地被列为全国绿色食品原料标准化基地。粮食生产连年丰收，均完成省下达的考评任务。

自主创新能力显著增强。五年共创建省级以上工程技术研究开发中心等创新载体22个，组建云浮石材研究院、物联网研究院等研发机构6所。获批省级以上科技项目374项，其中省部产学研合作项目99项；被批准为全省第一批产学研合作示范市，2010年获中国产学研促进奖，2011年被评为全国科技进步先进市。建成国家石材产品质量监督检验中心（广东）和五金制品、石材产品省级检验站，省级五金制品和石材专业标准化技术委员会先后落户。五年共有15个产品获广东省名牌产品称号，有8个特色产品成为国家地理标志保护产品；新增广东省著名商标25件、驰名商标和地理标志证明商标各2件。

（三）双转移取得新成效。

四大园区建设加快推进。佛山（云浮）产业转移工业园入围省示范性产业转移工业园，竞获扶持资金5亿元，并被认定为省高新技术产业开发区；佛山顺德（云浮新兴新成）产业转移工业园竞获省专业性产业转移工业园扶持资金1亿元；云浮循环经济工业园被认定为全省首批循环经济工业园；云浮双东环保工业园被省环保局批准为环保类专业园区。截至2011年底，四大园区累计投入基础设施建设资金47.5亿元，带动社会投入22.6亿元；入园项目368个，计划总投资724.3亿元。其中佛山（云浮）产业转移工业园累计投入基础设施建设资金26.6亿元，入园项目235个，计划总投资406亿元，建成投产项目182个。

招商引资和项目建设迅猛发展。五年共签约项目833个（含内外资），合同投资总额953.9亿元。其中2011年签订项目239个，合同投资总额462亿元，引资总额相当于“十五”时期的3.3倍。丰达、爱信精机、嘉吉、家乐福等世界500强、大型外资企业相继落户。2009—2011年，共确定210个项目为市重点工程项目，完成投资444.2亿元。其中2011年安排100项市重点工程项目，完成投资176.7亿元。

农村劳动力实现有序化技能化规模化转移就业。五年共转移农村劳动力25.6万人，其中转移到珠三角等地9.5万人；组织技能培训14.8万人次，培训后转移就业13.4万人。我市校企合作订单式等培训模式获上级充分肯定。

（四）交通基础设施建设实现新跨越。

五年共投入209亿元，是此前13年累计投入的3倍。全力推进以“七三三三一”工程为重点的交通基础设施建设。建成广梧高速（云浮段）、云浮新港，全面整治西江航道（云浮段）。云岑高速、阳罗高速、南广高铁、罗岑铁路、温氏都杨码头、云浮港都杨通用码头（煤码头）等均已动工建设；江罗高速已完成项目动工前期工作，汕湛高速清远清新至云浮新兴段省已同意开展项目业主招标。云肇轻轨、怀罗高速、罗信高速和云浮新港、都杨、温氏三大物流园前期工作进展顺利。升级改造国道324线（云浮段）及省道368线等一批公路，实现通镇、通村公路硬底化。截至2011年底，全市等级公路通车里程共7420公里，五年新增603公里，其中高速公路98.5公里；公路密度从2006年的每百平方公里87.7公里提高到95.4公里。

（五）人居环境建设创出新亮点。

城乡规划建设水平显著提高。启动云城、云安同城化和都杨新城区（城北新城区）建设，云六大道和河杨公路先后完成升级改造，沿江公路（六都至都杨段）基本贯通，新城快线（云杨公路）建设加快推进，初步搭起“一江三组团”城市发展路网架构。改造完善南山森林公园、市人民广场和市城区供水管网及部分主干道路。建成城市型慢行绿道80公里、郊野绿道300公里、山地自行车赛道6公里。创建宜居城镇16个、宜居村庄133条、宜居社区32个、生态文明村2819条；分别获省优秀城乡规划设计奖、宜居环境范例奖各1项，省宜居示范镇1个、宜居示范村庄8条。

电网、水利等基础设施建设日臻完善。五年共投入电网建设资金33.2亿元，其中农网改造15.8亿元，新建、改造10千伏及以上输变电线路2570公里；投入水利建设资金14.3亿元，完成13宗城乡防灾减灾、79宗中小型水库除险加固和惠及74.8万人的饮水安全工程。

生态建设和环境保护取得显著成绩。化学需氧量、二氧化硫排放量控制在省下达的目标以内。单位GDP能耗1.227吨标准煤，比2006年下降16.3%。全面完成集体林权制度改革任务，成功创建省林业生态市，森林覆盖率达67%。提前实现县县建成污水处理厂目标，城镇污水处理率达63.3%，城区生活垃圾无害化处理率达100%，饮用水源水质达标率100%。建成155条省级卫生村和14.8万户农村卫生厕。全面推行城乡垃圾统一收集清运。成功创建省卫生城市并启动创建国家卫生城市。

（六）基本公共服务均等化迈出新步伐。

社会保障水平不断提高。五年共投入民生事业134.2亿元，占财政支出59.8%。城镇新增就业16万人，登记失业率控制在3.5%以内。建成保障性住房3120套。基本医疗保险实现城乡一体化，社会养老保险覆盖城乡居民。城乡困难群众最低生活保障、农村“五保”和城镇“三无”人员供养、特殊困难群众大病医疗救助等制度进一步健全。我市成为全国金保工程建设示范市。

扶贫开发“双到”工作成效显著。累计投入帮扶资金5.1亿元，改善贫困村生产生活条件，发展集体经济项目450个，惠及贫困户3.2万户，实现“三年任务两年基本完成”目标。2010年省考评获“优”等次。

教育、文化、卫生、体育等事业全面发展。城乡免费义务教育全面实施，学前教育、特殊教育、高中阶段教育和高等教育加快发展。高中阶段教育毛入学率达到85%以上，通过省“普高”验收。高考上本科人数逐年增加，高等教育在校生达到4.6万人。校舍安全工程建设有序推进。市教育园区建设加快推进，市中专新校区（首期）基本建成。教师工资福利待遇“两相当”全面落实，“代转公”问题全面解决。新建市图书馆、博物馆、档案馆等一批公共文化设施。农村电影放映、农家书屋等文化惠民工作居全省前列。创建“广东省禅文化创意产业园”和“广东省石艺创意产业园”。完成全国第三次文物普查和第二轮地方志编修。基本完成基层医疗卫生机构综合改革，率先实施国家基本药物制度。建设市人民医院新院，扩建市中医院，完成 20间镇（街）卫生院改造和829间农村卫生站规范化建设。成功创建全国基层（社区和农村）中医药工作先进单位、全省首个农村中医工作先进市，创建4个全国农村中医工作先进县（市、区）。人口和计划生育工作进入全省先进行列。成功组织第16届亚运会火炬传递云浮站活动，承办2011年全国青少年（男子）曲棍球锦标赛和举办第三、第四届市运会。参加第13届省运会和第6届省残运会获金牌总数分别名列全省第12位、第13位。成功创建并连获省双拥模范城。国防建设和民兵预备役、统计、物价、人事人才、知识产权、地震、民族宗教、档案、外事侨务、港澳事务、台务、人民防空、消防、保密、气象、侨联、文联、社科联、科协、工商联、红十字会、贸促会、新闻出版以及驻市外机构等工作也取得新成绩。

平安和谐云浮建设扎实推进。认真落实领导接访和包案制度，加大社会治安综合治理力度，严厉打击各类违法犯罪活动，全面建成县镇村综治信访维稳中心，圆满完成奥运会、亚运会、亚残运会和大运会举办期间的安保和维稳任务，全市社会大局保持稳定。加大打私、打假、打击传销和食品药品安全专项整治力度，市场秩序不断规范。应急管理机制进一步完善，预防和应对突发事件能力有效提升。切实抓好安全生产，有效防范了重特大安全事故的发生。全面推进和谐劳动关系示范区创建活动，劳动者合法权益得到切实保障。

人民生活明显改善。2011年城镇居民人均可支配收入16367元、农村居民人均纯收入7553元,分别是2006年的1.6倍和1.7倍。城乡居民储蓄存款余额达383.9亿元。市政府承诺为群众办的“十件民生实事”按计划完成。

（七）体制机制改革闯出新路子。

实施主体功能扩展、三网融合、三规合一、美好环境与和谐社会共同缔造行动等工作创新，获上级肯定和社会广泛关注。农村综合改革、农村金融改革、“以奖代补”推动项目建设等经验在全省推广。我市被国务院批准全国三网融合试点城市，被国家有关部门批准为全国农村改革试验区、电子商务三网融合创新示范城市，被省批准为全省三规合一试点市、农村改革发展试验区、农村信用体系建设示范区、村级公益事业建设“一事一议”财政奖补工作示范市，以及三网融合双向互动数字电视应用、远程教育应用、远程医疗会诊和智慧城市试点市，云浮三网融合应用示范产业基地列为全省首批战略性新兴产业基地。

（八）政府施政能力实现新提升。

认真执行市人大及其常委会各项决议并报告工作，主动向市政协通报情况，广泛听取民主党派、工商联、无党派人士和各人民团体意见。五年共办理人大代表建议469件、政协委员提案859件。全面完成“五五”普法任务。清理审批事项294项。完成新一轮市县政府机构改革，全面开展富县强镇、简政强镇事权改革和事业单位分类改革。各级行政服务中心和行政电子监察系统进一步完善，市、县两级工程建设领域项目公开平台全面开通。率先在全省建立“农村廉政直通车”制度。市政府班子在省党风廉政建设责任制考核中连续四年被评为“好”的等次。

过去五年取得的成绩来之不易。这是省委、省政府和市委正确领导的结果，是市人大及其常委会、市政协和社会各界监督支持的结果，是全市上下在复杂困难环境中抓准机遇、化危为机的结果，是历届市委、市政府班子艰苦创业、打下良好基础的结果。在此，我谨代表市人民政府，向全市广大干部群众，向所有关心支持我市发展的各级领导和港澳同胞、台湾同胞、海外侨胞，驻云浮部队和武警、消防官兵，各民主党派、工商联、人民团体、社会各界人士，表示最衷心的感谢和最崇高的敬意！

各位代表，回顾过去五年的工作，我们得到许多有益的启示：一是必须坚持科学发展不动摇，敢于探索科学发展新路，敢于自加压力，迎难而上，才能不断创出工作新业绩、实现新突破。二是必须坚持加快发展不动摇，抢抓发展机遇，推进新型工业化、新型城市化和农业现代化“三化”融合发展，才能从根本上改变云浮相对欠发达的状况。三是必须坚持体制机制创新不动摇, 调动一切积极因素，营造和谐发展环境，才能不断增强加快发展的活力和动力。四是必须坚持执政为民不动摇，实实在在为老百姓谋福祉，才能不断赢得人民群众的信任和支持。五是必须坚持依法行政不动摇，大力优化政务环境，才能不断提高推动科学发展的能力。

同时，我们也必须清醒地认识到，经济总量小、发展不充分仍然是我市面临的主要矛盾。主要是产业发展水平不高，还没有形成一批支撑经济快速发展的优势产业集群；基础设施建设滞后，城镇化水平较低，尤其是中心城区集聚辐射带动能力不强；公共事业发展相对滞后；政府服务效能有待提高。对这些困难和薄弱环节，我们将采取切实措施，认真予以解决。

今后五年的奋斗目标和总体要求

今后五年，是我市实现经济社会跨越赶超、科学崛起的重要时期，我们面临重大发展机遇。从国际看，重振全球经济增长已成为世界各国广泛共识，有利于我市扩大对外开放、拓展发展空间。从国内看，坚持以科学发展为主题，坚持以转变经济发展方式为主线，继续实施积极的财政政策和稳健的货币政策，着力扩大内需促增长，我省加快实施双转移和区域协调发展战略，必将为我市发展提供强有力的支持。从我市看，已形成加快发展的良好态势，尤其是随着园区和交通等基础设施的不断完善，后发优势日益凸显，这为我市加快融入珠三角、加快发展迎来难得的机遇。只要我们抢抓机遇、勇于进取、务实工作，就一定能够在新的起点上实现新跨越。

今后五年的总体要求是：坚持以邓小平理论和“三个代表”重要思想为指导，深入贯彻落实科学发展观，全面落实市第五次党代会工作部署，围绕“跨越赶超、科学崛起”主题和建设幸福云浮目标，大力实施“集聚带动、转型拉动、创新驱动、统筹推动”发展战略，全面建设全省农村改革发展试验区、循环经济和人居环境建设示范市、广东富庶文明大西关，努力开创云浮科学发展、跨越发展新局面。

今后五年的奋斗目标是：全市地区生产总值、全社会固定资产投资总额、规模以上工业增加值、地方财政一般预算收入年均分别增长15%、28%、23.5%和20%以上，每年增速高于全国全省平均水平，位居全省前列；全市科学发展水平、人民幸福指数、社会事业发展水平在全省排位不断前移；产业结构更加优化、城乡建设更加协调、生态环境更加良好。

根据上述总体要求，实现今后五年奋斗目标，必须在“六个全面提升”上下功夫：

（一）全面提升基础设施建设水平。按照加快融入珠三角的要求，高起点规划建设交通网络，重点围绕中心城区、县（市）城区、四大园区、特色产业集聚带，全面推进高速公路、高速铁路、城际轻轨、西江航道和港口码头建设，加快各类交通运输站场和县镇村公路建设与改造，实现与珠三角、粤西以及大西南地区的快速通达。加强水利基础设施建设，提高城乡防灾减灾能力和水资源利用率。加快能源、信息等基础设施建设，为经济社会发展提供强有力支撑。

（二）全面提升产业集聚发展水平。坚持以转变经济发展方式为主线，做优产业结构，做大产业集群，做强企业实力，加快新型工业化步伐；坚持以农民增收为核心，以改善农村发展环境为重点，加快农业现代化进程，推动传统农业向现代农业、传统农业大市向现代农业强市跨越；坚持优先发展现代服务业，大力发展生产性服务业，提高服务业增加值占地区生产总值比重；大力发展各具特色的地方经济，加快壮大县域经济。

（三）全面提升城乡建设水平。突出宜居城乡建设，加快新型城市化进程。坚持绿色、智慧、包容和以人为本发展理念，高起点、高标准、高水平抓好城乡发展规划，加快建设宜居的生态绿城、宜业的创业新城、平安的和谐之城，努力把云浮建设成为有特色、有品位、值得作为故乡的宜居宜业平安幸福之城。

（四）全面提升民生保障水平。坚持以人为本，切实解决人民群众最关心、最直接、最现实的利益问题。坚持教育优先发展，提升教育质量，加快创建教育强市。深化医药卫生体制改革，完善医疗卫生服务体系。扩大城乡社会保险覆盖面，完善惠及全民的社会保障体系。加强农村劳动力培训和转移就业工作，加大扶贫开发力度，切实提高城乡居民收入，让人民群众共享发展成果。

（五）全面提升文化强市建设水平。大力挖掘、传承和弘扬优秀传统文化，提高公民道德水平。积极举办和参与禅宗文化交流活动，努力建设“世界禅宗文化思想高地”。加快构建覆盖城乡的普惠型公共文化服务体系，让群众文化惠及百姓。推动文化资源向产业资源转化，加快文化产业基地建设，促进文化与经济融合发展。

（六）全面提升社会建设水平。以人民群众满意为目标，全面建设服务、责任、法治和廉洁高效政府。加快推进基本公共服务均等化，全面发展社会各项事业。加强和创新社会管理，深入推进法治云浮建设，努力维护社会和谐稳定。

各位代表，市第五次党代会和人代会的相继召开，标志着云浮发展新的航程已经开启。只要全市人民团结一致、奋力拼搏，就一定能圆满实现今后五年经济社会发展各项目标任务，开创更加美好的明天。

2012年工作安排

2012年是新一届政府的开局之年，也是为实现全市经济社会跨越赶超、科学崛起打好基础的关键一年。做好今年工作责任重大。我们要全面贯彻落实党的十七届六中全会、中央经济工作会议、省委十届十一次全会精神和市第五次党代会工作部署，聚精会神搞建设，一心一意谋发展，全力推动各项工作有效落实。

2012年主要预期目标是：地区生产总值增长15%；地方财政一般预算收入增长22%；社会消费品零售总额增长18%；全社会固定资产投资总额增长30%；城镇居民人均可支配收入增长12%；农村居民人均纯收入增长11.9%；单位GDP能耗下降3.89%，主要污染物排放量控制在省考核指标以内；人口自然增长率控制在7‰以内；城镇登记失业率控制在3.5%以内；居民消费价格指数控制在4%以内。

实现今年目标，我们必须抓好以下八方面工作：

（一）加快推进转型升级，做优存量经济。

加快工业集聚发展。全力推进“千亿工业”大会战，大力实施工业企业成长工程，推动“五大”特色产业加快转型升级。加快“中国·云浮国际石材产业城”、中材水泥、华润水泥、青洲水泥、云浮风电等项目和新兴县不锈钢产业园、省市共建先进制造业（硫化工）产业基地建设，力促华润西江发电厂、云浮核电站（中广核、中电投）、云浮电厂D厂、粤泷电厂二期、郁南抽水蓄能发电等电力能源项目立项申报取得突破。制定石材等产业的行业标准，力争打造一批国内外知名品牌。推进工业化与信息化融合，利用高新技术、先进适用技术和信息化改造提升机械制造、纺织服装、农产品深加工等传统产业，加快发展生物制药、电子信息、电池新材料等新兴产业。发挥云浮循环经济工业园、云浮双东环保工业园示范带动作用，加快发展循环经济，打造一批环境友好型特色产业。年内继续培育一批规模以上工业企业，规模以上工业增加值增长29%以上。

发展壮大现代服务业。制定生产性服务业发展规划，大力引入生产性服务业项目和培育本土龙头服务企业。发挥生产性服务业在传统制造业转型升级过程中的作用，加快发展信息、金融、会展、咨询、物流等现代服务业。完善政银企合作机制，落实与四大商业银行和国开行授信融资协议，保持信贷资金较快增长。着力开发禅宗文化、温泉、原始生态森林、生态观光农业、农家乐等旅游资源，力争全年实现接待游客965万人次、旅游总收入105亿元。办好第九届中国（云浮）国际石材科技展览会、第三届广东（云浮）石文化节。加快宝利来、华立、远大等五星级酒店和东方时代广场、家乐福等城市商贸综合体项目及各县（市、区）房地产项目建设。推动三网融合应用、物联网、文化创意、健康休闲等战略性新兴产业加快发展。

加快发展现代农业。推进国家和全省农村改革发展试验区建设，大力实施现代农业提升工程，推动农村发展、农业增效、农民增收。推进农民专业合作社示范县建设，发挥温氏集团龙头带动作用，打造国家级畜牧业产业化示范中心，进一步提高农业产业化水平。年内新发展省级农业龙头企业 2家、市级农业龙头企业 10家、县级农业龙头企业15家、农民专业合作社50个。加强农业现代化示范区和现代农业园区建设，加快有机食品、绿色食品和无公害农产品生产基地建设，年内新创建优势特色农产品示范基地5个。抢抓国家推动水利改革发展机遇，精心组织实施一批重大水利工程。加快佛山(云浮)产业转移工业园防洪治涝、南山河城区防洪和罗定市引沙灌区等工程建设，年内动工建设8宗中小河流治理项目、完成16宗小型水库除险加固和30宗灌区改造工程。推进村村通自来水工程。加快中低产农田改造，年内建设1.8万亩现代标准农田，确保粮食种植面积稳定在180万亩、总产66万吨以上。

加快发展外源型经济。加快加工贸易企业转型升级，加大对重点行业和龙头骨干企业出口的支持。推动外商投资企业、加工贸易企业扩大内销。加快云浮海关业务技术综合楼等口岸服务平台建设，进一步优化通关环境。加强与港澳台经贸合作，积极开拓东盟、非洲、俄罗斯等新兴国家贸易市场，拓展对外开放广度和深度。组织参加第111、112届广交会、第四届广东外商投资企业产品（内销）博览会、2012年粤港澳等境内外经贸合作交流活动。力争全年进出口总额和出口总额分别增长12%以上。

加强自主创新。发挥云浮高新区科技企业孵化器、国家和省级工程技术研究开发中心等创新载体以及国家石材产品质量监督检验中心（广东）等平台的作用，加大创新型人才引进和培养，加强三网融合、物联网、生物制药等领域关键技术的研究、开发和应用，推动新兴产业创新发展。进一步深化产学研合作，重点扶持具有自主知识产权、自主品牌的创新型企业发展，培育一批高新技术企业。

（二）加快推进双转移，做大增量经济。

加快四大园区建设。按照产业集聚发展、土地集约利用、生活设施集中配套的要求，进一步完善园区基础设施、征地报批和公共配套服务设施，提升园区产业集聚能力，促进产业向园区集聚，形成优势特色产业集群。创新园区管理体制，优化园区发展软环境。年内四大园区新动工和建成项目投资额均要实现增长35%以上，其中佛山（云浮）产业转移工业园和佛山顺德（云浮新兴新成）产业转移工业园要达到40%以上，成为全市主要经济增长点。

提升招商引资质量。加强产业招商、平台招商和重点招商，力争年内引进投资额10亿元以上项目10个，其中世界500强或大型外资企业2家，大型央企2家、龙头民企6家。坚持引进项目与引进技术、管理、人才相结合，鼓励外资更多投向传统优势产业和战略性新兴产业，提高利用外资水平。力争年内合同利用外资和实际利用外资分别增长12%以上，确保招商活动签约项目履约率达到70%以上。

加快重点工程项目建设。健全抓落实责任机制，提高服务水平，全力抓好总投资1057.2亿元、年内计划投资191.8亿元的100项市重点工程项目建设，进一步增强发展后劲。

加快农村劳动力转移就业。以基层公共服务平台建设为突破口，以技能培训为抓手，以劳务对接为依托，推进农村劳动力有序化技能化规模化转移就业。推进技工院校与产业转移园区企业对接，全面推行校企合作订单式培训、技能下乡培训、结对培训和特色培训等培训模式，为我市实施双转移战略提供大批的现代产业工人。年内培训农村劳动力3.7万人，新增转移就业6万人。

（三）加快推进交通基础设施建设，增强区位竞争力。

加快“七三三三一”等交通工程建设。加快云岑、阳罗等高速公路建设，年内建成云岑高速双凤至罗定段，动工建设江罗高速。推动南广高铁全面复工、罗岑铁路加大投资力度。加快温氏都杨码头、云浮港都杨通用码头（煤码头）和云浮新城物流园、云浮新港物流园等物流产业基地建设。继续开展西江航道整治和界首至南江口段文明样板航道建设。推进怀罗、罗信高速和云肇轻轨、都杨西江特大桥等项目前期工作，落实汕湛高速清远清新至云浮新兴段项目业主。继续谋划申报一批重大交通基础设施项目。

加快市域交通网络建设。推进国道、省道和县乡公路升级改造，加快农村公路建设。年内完成国道324线河口至安塘段改线、沿江公路（六都至都杨段）扩建和省道276线、113线大修改造工程。加快推进新城快线（云杨公路）和市新城区客运站、货运中心等交通运输站场建设。发展公交事业，推进城乡客运一体化。

（四）加快推进宜居城乡建设，增强发展承载力。

做好城乡发展规划。按照三规合一要求和“一江三组团”城市发展格局，高标准修编完善城乡、城市总体规划和各类控制性详细规划，完善空间地理信息共享平台，全面优化空间布局。年内重点开展城西新区、城东片区和城北新城区发展规划编制和全市农林水土及生态环境建设规划编制。加强规划管理，严厉打击违法违章建设行为。加强土地储备，加快推进“三旧”改造和市城区石材企业搬迁工作，加大闲置土地处置力度，提高土地集约节约利用水平。

完善城市服务功能。完善中心城区、县城和中心镇的交通、供电、供水、污水处理等公共服务设施以及教育、医疗、旅游、商业等综合服务功能。推进市城区道路贯通工程，缓解交通拥堵问题。推进市城区应急备用水源及都杨西江水厂建设。完成云城区河口街市政综合开发建设，启动蟠龙天湖景区改造和市体育公园等市民活动休闲场所建设。加强城市综合管理，创建智慧城市，提高城市信息化、智能化水平。巩固提升省卫生城市成果，推进创建国家卫生城市工作。

加强生态建设和环境保护。推进“一城三江三线”生态景观林带建设。实施林业重点生态工程和生态文明“万村绿”工程，创建省园林城市。年内造林19万亩，绿化村庄100条。继续创建一批名镇、名村和宜居城镇、宜居村庄（社区）。健全节能减排目标年度考核机制，严格执行主要污染物排放总量控制和前置审核制度，狠抓重点流域、区域、行业污染治理，确保完成节能减排目标任务。

（五）加快推进文化强市建设，增强发展软实力。

着力提升禅宗文化影响力。深入开展禅宗文化研究、挖掘，组织举办禅宗文化论坛等研讨活动，完善禅文化旅游配套服务设施，加强与日本、朝鲜、韩国等国家的交流，提升禅宗文化影响力。

着力发展群众文化。完善城乡公共文化服务网络，推进乡镇综合文化站、村（社区）文化室、农家书屋等文化场所建设。继续开展农村电影放映、送戏下乡等活动，丰富群众文化生活。鼓励支持文艺创作。深入挖掘和整理南江文化。加强文物和非物质文化遗产保护。

着力发展文化产业。大力促进文化与经济融合发展，推进禅文化创意产业园和石艺创意产业园建设，年内在文化产业项目引进和建设上实现新突破。加快文化改革发展，全面完成经营性文化单位改革。

（六）加快推进社会事业建设，增强发展凝聚力。

优先发展教育事业。加快发展学前教育，推行在小学增设附属幼儿园，年内全市各镇（街）建设1所以上规范化中心幼儿园，基本普及学前三年教育。注重发展特殊教育。大力推进义务教育规范化学校建设。加快薄弱高中改造和中职学校建设，提升高中阶段教育水平。加强校园安全和校车安全管理。加快市教育园区和华立学院建设，年内建成市技工学校新校区（一期）。着力解决市中心城区优质学位不足问题，扩建新建幼儿园、小学、中学各2所。鼓励和支持创建教育强镇、教育强县，启动创建教育强市工作。

完善城乡就业服务体系。实施全民技能提升储备计划，开展公益性职业技能培训，促进高校毕业生、返乡农民工、就业困难人员就业。倡导和鼓励自主创业，推动以创业带动就业。规范企业劳动用工行为。加快镇（街）人力资源社会保障服务所建设，组织各类大型招聘会，拓展网上就业服务。年内新增城镇就业岗位3.1万个，失业人员实现再就业0.9万人，免费创业培训0.15万人。

完善社会保障制度。加强社会保险扩面征缴和市级统筹，做好职工医疗保险、城乡居民基本医疗、社会养老保险与城乡医疗救助等制度的统一衔接。完善困难群众社会救助体系和残疾人服务体系，切实保障农村贫困家庭、城镇困难家庭、老弱孤寡残等困难群体的基本生活。抓好扶贫开发“双到”工作，全面完成脱贫目标任务。发展社会福利和慈善事业。健全覆盖社区、乡镇的平价商店网络。建设保障性住房1100套。

加强医疗卫生建设。完善基层医疗卫生机构综合改革工作，加快推进公立医院改革。继续抓好市人民医院新院和市妇幼保健院新院建设。完善市、县、镇、村四级医疗卫生服务体系和医疗卫生机构标准化信息化建设，年内完成30间基层医疗卫生机构（卫生院和社区卫生服务中心）升级改造，完成1.5万户农村改厕。全面落实国家基本药物制度。加大卫生监督和重大传染病防治工作力度。加强中医药工作，巩固全国基层（社区和农村）中医药工作先进单位成果。引导民营医疗机构健康发展。

加强和创新社会管理。加强流动人口、娱乐场所、出租屋管理，严厉打击黑恶势力、“两抢一盗”等严重刑事犯罪活动。继续抓好综治信访维稳“三级平台”建设，完善“大排查”、“大调解”、“大接访”等工作机制，切实预防和妥善处置群体性事件。继续探索完善三级理事会。加强社会建设信息化工作，完善应急管理机制。严格落实安全生产“一岗双责”，强化各级政府监管责任和企业主体责任，防范和遏制重特大安全事故发生。严厉打击制假售假、走私行为，加强食品药品安全管理和无证无照经营综合治理，规范市场秩序。

统筹其他各项事业发展。加强国防教育、人民防空和民兵预备役工作，做好军队转业人员安置工作，巩固省双拥模范城成果。加强流动人口计划生育工作，稳定低生育水平，综合治理出生人口性别比偏高问题。深入推进“六五”普法，加强法律援助和司法救助。切实维护妇女儿童合法权益。认真做好统计、民族宗教、外事侨务、国资、气象、方志、档案、对台、老龄等各项工作。

提高财政保障能力。大力培育和扶持重点财源税源，加强税收征管和非税收入管理，确保财政收入较快增长。优化财政支出结构，加强财政资金的预算管理、使用监督、责任审计和绩效考评。加大对教育、文化、卫生、体育和就业、医疗、养老、保障性住房等民生事业投入，提高民生保障水平。

各位代表，我代表市人民政府承诺，今年继续集中力量为人民群众办好扶贫、就业、社保、住房、教育、医疗卫生、文化体育、物价、农田水利和宜居环境建设等方面的十件实事。

（七）加快推进改革创新，增强发展动力。

推进体制改革。着力抓好培育和规范管理社会组织、深化住房保障制度、深化医疗保障制度等三大重点领域和关键环节的改革工作。深化户籍制度改革，完善土地流转机制，探索解决农村居民融入城市发展的有效途径。深化富县强镇、简政强镇事权改革和事业单位分类改革。推进云城、云安深化行政管理体制改革试点工作。

推进主体功能区建设。完善各县（市、区）主体功能扩展规划。全面实施与主体功能区建设相配套的财政保障机制和政绩考核机制。继续深化农村综合改革、农村金融改革工作。

推进信用云浮建设。完善市、县征信中心建设，创建一批信用村、信用企业，逐步建立覆盖城乡居民、各行各业和服务全社会的征信体系。加强社会诚信宣传教育，营造“讲信用、守信用”的良好社会环境。

推进美好环境与和谐社会共同缔造行动。全面实施村级公益事业建设“一事一议”财政奖补政策，推行自然村（居民小组）竞争性“以奖代补”项目。探索外出乡贤在社会管理、公益事业和经济发展中发挥作用的新途径，推进新农村建设。全面开展精神文明创建活动，推进创建省文明城市工作。

（八）加快推进政务环境建设，增强政府服务发展能力。

提高依法行政水平。加强执法公开、政务公开、行政复议和行政应诉工作，依法科学民主决策，提高依法行政能力。完善向人大及其常委会报告、向政协通报工作制度，认真落实和执行人大决议、决定，自觉接受人大的依法监督和政协的民主监督，切实提高办理人大建议、政协提案质量。

提高行政效率。创新政府管理方式，推进电子政务和网上审批。加强政风建设，转变机关作风，把更多精力放在服务企业、监管市场和解决民生问题上。推进全省“工程领域项目信息公开和诚信体系建设”、“地方政府及政府机关绩效管理”试点示范市创建工作。健全机关效能监察机制，严肃查处政令不畅、办事效率低等问题。健全重点工作领导挂帅和检查督促机制，围绕重大决策、重要事项、重点工程加强督查督办并严格问责。对落实科学发展观、建设幸福云浮和经济发展、重点工程项目建设等工作实行考核。

加强干部队伍建设。推进廉政建设，确保反腐倡廉各项措施落到实处。深化领导干部经济责任审计，规范权力运行。切实纠正部门和行业不正之风，严肃查处损害企业和群众利益的行为。加强公务员培训，建设政治坚定、业务精通、清正廉洁、作风优良的公务员队伍。

各位代表，发展的蓝图令人鼓舞，崇高的使命催人奋进。让我们在中共云浮市委的坚强领导下，齐心协力，开拓创新，真抓实干，为实现跨越赶超、科学崛起，建设幸福云浮作出新的更大的贡献！
